# Diagnostic value of LGE and T1 mapping in multiple myeloma patients’heart

**DOI:** 10.1186/s12872-024-03895-y

**Published:** 2024-04-27

**Authors:** Qian Cui, Jing Yu, Xihong Ge, Guangfeng Gao, Yang Liu, Qiang He, Wen Shen

**Affiliations:** 1 The First Central Clinical School, Tianjin Medical University, Tianjin, China; 2 Department of Radiology, Tianjin First Central Hospital, School of Medicine, Nankai University, No.24 Fukang Road, Tianjin, 300192 China; 3 Department of Cardiology, Tianjin First Central Hospital, School of Medicine, Nankai University, Tianjin, China

**Keywords:** Cardiovascular magnetic resonance, Late gadolinium enhancement, T1 mapping, Heart failure, Multiple myeloma

## Abstract

**Background:**

Unidentified heart failure occurs in patients with multiple myeloma when their heart was involved. CMR with late gadolinium enhancement (LGE) and T1 mapping can identify myocardial amyloid infiltrations.

**Purpose:**

To explore the role of CMR with late gadolinium enhancement (LGE) and T1 mapping for detection of multiple myeloma patients’heart.

**Material and methods:**

A total of 16 MM patients with above underwent CMR (3.0-T) with T1 mapping (pre-contrast and post-contrast) and LGE imaging. In addition, 26 patients with non-obstructive hypertrophic cardiomyopathy and 26 healthy volunteers were compared to age- and sex-matched healthy controls without a history of cardiac disease, diabetes mellitus, or normal in CMR. All statistical analyses were performed using the statistical software GraphPad Prism. The measurement data were represented by median (X) and single sample T test was adopted. Enumeration data were represented by examples and Chi-tested was adopted. All tests were two-sided, and *P* values < 0.05 were considered statistically significant.

**Results:**

In MM group, LVEF was lower than healthy controls and higher than that of non-obstructive hypertrophic cardiomyopathy group, but without statistically significant difference (%: 49.1 ± 17.5 vs. 55.6 ± 10.3, 40.4 ± 15.6, all *P* > 0.05). Pre-contrast T1 values of MM group were obviously higher than those of healthy controls and non-obstructive hypertrophic cardiomyopathy group (ms:1462.0 ± 71.3vs. 1269.3 ± 42.3, 1324.0 ± 45.1, all *P* < 0.05). 16 cases (100%) in MM group all had LGE.

**Conclusion:**

LGE joint T1 mapping wider clinical use techniques and follow-up the patients’disease severity.

## Introduction

Multiple myeloma is characterized by dysplasia of bone marrow plasma cells with monoclonal immunoglobulin or light chain (M protein) overproduction. Because the M protein misfolds to form amyloid protein, its abnormal deposition leads to amyloidosis. Therefore, the presence of any M protein may cause amyloidosis. According to statistics, about 20% of myeloma can be combined with amyloidosis. The most important damage of amyloidosis caused by multiple myeloma is myocardial amyloidosis. The pathogenesis of this kind of patients is complex, and the clinical manifestations of heart damage are diverse, heart failure will occur when involving the heart, early diagnosis of wheather myocardial was involved is important. Amyloidosis is a rare diverse condition caused by the pathologic extracellular deposition of abnormal insoluble proteins throughout the body. It may exist as a primary disease or, more commonly, may be secondary to a wide array of pathologic conditions ranging from chronic infection or inflammation to malignancy. Hereditary forms also exist. Based on the structure of protein deposits, over two dozen subtypes of amyloidosis have been described, with light-chain (AL) and transthyretin (ATTR) being the most common subtypes, Multiple myeloma often causes light-chain (AL) myocardial amyloidosis [1] and often presents as a challenging diagnostic dilemma [2, 3]. A single organ or multiple organ systems may be affected. In the chest, the lungs, mediastinum, pleura, and heart may be involved [4]. Approximately 50% of patients with AL amyloidosis develop cardiac involvement [5], in contrast to only approximately 2% of patients with AA amyloidosis [6]. Cardiac amyloidosis (CA) is a challenging and underdiagnosed cause of heart failure [7, 8]. Cardiac magnetic resonance (CMR) imaging has grown rapidly in clinical application over the past several decades, and numerous clinical and experimental studies have validated CMR imaging as a useful noninvasive tool for diagnosing and managing cardiovascular disease. CMR imaging now plays a pivotal role in cardiac morphologic and functional assessment and tissue characterization, allowing for evaluating various pathologic conditions ranging from myocardial infarction and ischemic or non-ischemic cardiomyopathy to cardiac involvement in systemic diseases such as amyloidosis and sarcoidosis. CMR with late gadolinium enhancement (LGE) identifies myocardial amyloid infiltration as a characteristic pattern of global sub-endocardial or transmural LGE coupled with abnormal myocardial and blood-pool gadolinium kinetics [9–13]. Traditional LGE MR images are most useful for evaluating focal diseases since they allow for the use of normal myocardium as a standard of reference and the detection of an enhancement pattern. If gadolinium uptake is uniform, diffuse fibrosis may go undetected on qualitative images; T1 mapping is sensitive to myocardial edema and diffuse fibrosis. To our knowledge, T1 mapping for diffuse fibrosis quantification has been validated relative to histologic findings only in small numbers of patients with hypertrophic cardiomyopathy, aortic valve disease [14, 15], and in the postoperative cardiac transplantation setting [16]. Native T1 mapping has emerged as a potentially useful diagnostic CMR technique for the identification of both AL and ATTR CA without recourse to contrast agents [17, 18]. Abnormal native T1 mapping and ECV were associated with higher all-cause mortality in CA. Therefore, both quantitative techniques are valuable and should be considered in all patients undergoing MRI with suspected or confirmed CA [19, 20]. This study aims to determine the value of CMR in diagnosing and evaluating myocardial amyloidosis in MM patients by comparing differences between non-cardiac history, non-obstructive hypertrophic cardiomyopathy using LGE and T1 mapping.

## Material and methods

### Study population

CMR was performed in patients diagnosed with MM as having heart failure suspected myocardial involvement between May 2020 and November 2022. A total of 16 MM patients with renal biopsy as having AL amyloidosis (M:F = 9:7; mean age, 60.0 ± 8.9 years) underwent CMR (3.0-T) with T1 mapping (Native and post-contrast) and LGE imaging. In addition, We enrolled participants admitted for non-obstructive hypertrophic cardiomyopathy confirmed by cardiology department, 26 patients with non-obstructive hypertrophic cardiomyopathy (M:F = 16:10, age, 51.7 ± 12.4 years) and 26 healthy volunteers (M:F = 16:10; mean age, 49.9 ± 18.0 years) without a history of cardiac disease, diabetes mellitus, or normal in CMR were compared. Every participant agreed to undergo a hematocrit blood test within 24 h before CMR scan and informed consent was obtained from all subjects and/or their legal guardian(s).

The inclusion criteria of HCM group: a. Basal LV wall thickness ≥ 15 mm, b. ratio of septal thickness to thickness of inferior wall at midventricular level > 1.5, c. Peak gradient at LVOT or mid LV cavity < 30 mm Hg, d. no contraindications to cardiac MRI; the inclusion criteria of normal controls: without a history of cardiac disease, diabetes mellitus, or normal in CMR; the exclusion criteria of HCM group and normal controls: a. previous cardiac surgery, b. moderate to severe valvular heart disease, c. any permanent implanted device, d. Apical HCM, Obstructive HCM et. al any other cardiomyopathy. And there are 9/26 HCM paitents admission for heart failure, the LVEF is lower than 20%.

### Follow‐up

The study end-point was defined as the occurrence of cardiac death, heart transplantation, and hospitalization due to cardiovascular events. Follow-up information was obtained from in-person or telephone interview at 2-month intervals. Time to event was defined as the duration from the date of the CMR scan to an event. When had a treatment would review with native T1 mapping. The information from follow-up CMR acquisition as cardiac function (LVEDV and LVESV, LVEF and mitral valve regurgitation), cardiac morphological indicators (left ventricular wall thickness and left ventricular mass (LVM), native T1 values.

### CMR acquisition

CMR was performed on a 3.0 T MRI system (Ingenia, Philips Medical Systems, Best, the Netherlands) with an 16-channel cardiac phased-array coil.

Along with long-axis planes (two-, three-, and four- chamber views), a stack of short-axis single-shot balanced standard steady-state in free-precession sequence images from apex to basal were collected. The imaging parameters were as follows: field of view, 300 mm × 300 mm; voxels, 2 mm × 2 mm × 8 mm; repetition time, (3.0–3.2) ms; echo time, (1.5–1.6) ms; sense factor, 1.8; minimum inversion time, 105 ms; and flip angle, 45°. Cine CMRI was performed using a steady-state freeprecession sequence. Fat-saturated and T2-weighted images were obtained to allow differentiation among subepicardial LGE, epicardial fat, and pericardial effusion.

T1 mapping was performed for all participants in 3 slices with basal- ventricular, mid-ventricular and apex-ventricular short-axis view. For the native T1 mapping, MOLLI 5s(3s)3 s scheme was performed. A total dose of 0.2 mmol/kg gadopentetate dimeglumine injection was administered. For post-contrast, a 4s(1s)3s(1s)2s scheme was performed [10–15] min after the injection. The imaging parameters were as follows: field of view, 320 mm × 320 mm; voxels, 2 mm × 2 mm × 8 mm; sense factor, 1.5; minimum inversion time, 105 ms; and flip angle, 20°.

First-pass myocardial perfusion were performed multi-dynamic rapid capture of the heart in less than 1 min. The relative perfusion parameters were analyzed by observing the change of signal intensity of contrast agent through myocardium. The imaging parameters were as follows: field of view, 320 mm × 320 mm; voxels, 2 mm × 2 mm × 8 mm.

LGE images were performed along the long-axis and short-axis views using phase sensitive inversion recovery about [10, 11] min after the injection. The imaging parameters were as follows: field of view, 320 mm × 320 mm; voxels, 2 mm × 2 mm × 8 mm; repetition time, (6.0–6.2) ms; echo time, (3.0–3.1) ms; and TI, adjustedat that time.

### Image analysis

CMR analysis was performed inIntellispace Portal 7. Cine, T2WI, T1 mapping and contrast images (First-pass myocardial perfusion, LGE and post-T1mapping) were evaluated separately by 2 blinded observers. In brief, endocardial and epicardial borders were outlined on the short-axis cine images. Volumes, myocardial mass, and ejection fraction were derived by summation of epicardial and endocardial contours. For each segment, the extent of LGE was analyzed.

Observers to label: (1) cardiac function: LVEDV and LVESV, LVEF and mitral valve regurgitation; (2) cardiac morphological indicators: left ventricular wall thickness and left ventricular mass (LVM); (3) tissue features: T2 images myocardial signal, with or without perfusion defect and its position and scope, the presence of delayed enhancement and its position, shape, and scope, and the enhancement T1 values before and after, ECV; (4) accompanying signs such as pericardial effusion, and pleural effusion.

Myocardial T1 times were measured carefully in a global region of interest (ROI), including the whole ventricular wall; meanwhile, an ROI was drawn in the blood pool to measure blood pool T1 times. ECV was calculated as follows:

$$\textit{ECV}=\left(1-\text{hematocrit}\right)\times\left[\left(1/\mathrm m\mathrm y\mathrm o\;\mathrm p\mathrm o\mathrm s\mathrm t\;T1-1/\mathrm{myo}\;\mathrm{native}\;T1\right)/\left(1/\mathrm{blood}\;\mathrm{post}\;T1-1/\mathrm{blood}\;\mathrm{native}\;T1\right)\right]$$

### Statistics

To determine T1 values for the three groups, native T1 mapping was performed and the data computed from receiver operating characteristic curves (ROC). All statistical analyses were performed using the statistical software GraphPad Prism (version 9.0; GraphPad Software, San Diego, California, USA). The measurement data were represented by median (X) and single sample T test was adopted. Enumeration data were represented by examples and Chi-tested was adopted. A comparison was made between the healthy controls group and the MM group (P1), non-obstructive hypertrophic cardiomyopathy group and the MM group (P2). All tests were two-sided, and *P* values < 0.05 were considered statistically significant.

## Results

Joint inspection of the cardiac function indexes (Table 1): in MM group, LVEF was lower than healthy controls and higher than that of non-obstructive hypertrophic cardiomyopathy group, but without statistically significant difference ((%: 49.1 ± 17.5 vs. 55.6 ± 10.3, 40.4 ± 15.6, all *P* > 0.05); however, LVEDV and LVESV were significantly lower than healthy controls and non-obstructive hypertrophic cardiomyopathy group, statistically significant difference [LVEDV(ml/m^2^): 100.6 ± 33.8 vs.117.5 ± 25.9, 156.7 ± 49.8, LVESV(ml/m^2^): 53.6 ± 25.7 vs. 52.7 ± 16.3, 96.7 ± 50.3, all *P* < 0.05]. Native T1 values of MM group were obviously higher than those of healthy controls and non-obstructive hypertrophic cardiomyopathy group (ms: 1462.0 ± 71.3vs. 1269.3 ± 42.3, 1324.0 ± 45.1, all *P* < 0.05); and enhanced T1 values were less than those of the above two groups, but without statistically significant difference (ms: 479.3 ± 66.7 vs. 516.0 ± 61.1, 499.0 ± 65.9, all *P* > 0.05). LVM in MM group was obviously higher than that of healthy controls but less than that of non-obstructive hypertrophic cardiomyopathy group (g: 156.9 ± 50.6vs. 109.0 ± 27.0, 232.4 ± 89.8, all *P* < 0.05).

**Table 1:** MM and non-obstructive hypertrophic cardiomyopathy patients MRI results compared

	Healthy controls (*n* = 26)	Hypertrophic cardiomyopathy (*n* = 26)	Multiple myeloma (*n* = 16)	*P*_1_	*P*_2_
Age (years)	49.9 ± 18.0	51.7 ± 12.4	60.0 ± 8.9	0.18	0.36
Gender (M/F)	16/10	16/10	9/7	0.73	0.73
LVEDV (ml/m^2^)	117.5 ± 25.9	156.7 ± 49.8	100.6 ± 33.8	0.338	0.001
LVESV (ml/m^2^)	52.7 ± 16.3	96.7 ± 50.3	53.6 ± 25.7	0.995	0.004
LVEF (%)	55.6 ± 10.3	40.4 ± 15.6	49.1 ± 17.5	0.314	0.206
LVM (g)	109.0 ± 27.0	232.4 ± 89.8	156.9 ± 50.6	0.05	0.005
LVPWT (> 12 mm)	-	26(100%)	14(87.5%)	< 0.001	0.06
Mitralvalvular regurgitation	-	21(80.7%)	16(100%)	< 0.001	0.138
Perfusion defects	-	-	7(43.5%)	0.002	0.002
LGE	-	19(73.1%)	16(100%)	< 0.001	0.02
Native T1 (ms)	1269.3 ± 42.3	1324.0 ± 45.1	1462.0 ± 71.3	< 0.001	< 0.001
Enhanced T1 (ms)	516.0 ± 61.1	499.0 ± 65.9	479.3 ± 66.7	0.428	0.825
ECV (%)	27.7 ± 2.3	29.2 ± 4.3	45.7 ± 6.7	< 0.001	< 0.001
Pericardial effusion	-	-	6(37.5%)	< 0.001	< 0.001
Pleural effusion		-	9(56.3%)	< 0.001	< 0.001

*LVEDV* Left ventricular end-diastolic volume, *LVESV* Left ventricular end-systolic volume, *LVEF* left ventricular ejection fraction, *LVM* Left ventricular mass, *LVPWT* Left ventricular wall thickening, *ECV* extracellular volume

Cardiac morphological indexes of MRI results (Table 1): in MM group, 14 cases(87.5%) of patients had left ventricle end-diastolic maximal thickness (LVMT > 12 mm), including nine cases (56.2%) with right ventricular wall thickening together; all 26 (100%) patients of non-obstructive hypertrophic cardiomyopathy had left ventricular wall thickening, but none had right ventricular wall thickening.

Myocardial tissue characteristics (Table 1): LGE can be characterized by left ventricular patchy, subendocardial, occasionally transmural and not the typical abnormal LGE when amyloidosis involves the myocardium. A total of 16 cases (100%) in MM group all had LGE, including six cases of left ventricular wall patchy enhancement, six cases of left ventricular subendocardial diffuse enhancement, and three cases of transmural LGE; one case was not typical abnormal delay late enhancement; 19/26 cases (73.1%) in non-obstructive hypertrophic cardiomyopathy group had LGE, but segmental enhancement, Isolated or multiple patchy LGE at mid-wall and along the RV insertion points on the septum are the usual patterns of late enhancement in hypertrophic cardiomyopathy. LGE in HCM is usually localised in segments with the maximum LV wall thickness, confined to significantly different with MM’s cardiac amyloidosis enhancement pattern; and 26 cases of healthy controls had no LGE. There are 6/16 cases (37.5%) and 9/16 cases (56.3%) with pericardial effusion and pleural effusion in MM group.

Gadolinium clearance time in blood pools is approximately 5 min. Due to abnormal protein deposition, amyloidosis and blood pool clear faster, enhanced T1 values of MM group were obviously lower than those of healthy controls, but without statistically significant difference (ms: 479.3 ± 66.7vs. 516.0 ± 61.1, *P* > 0.05).

**Fig. 1:**
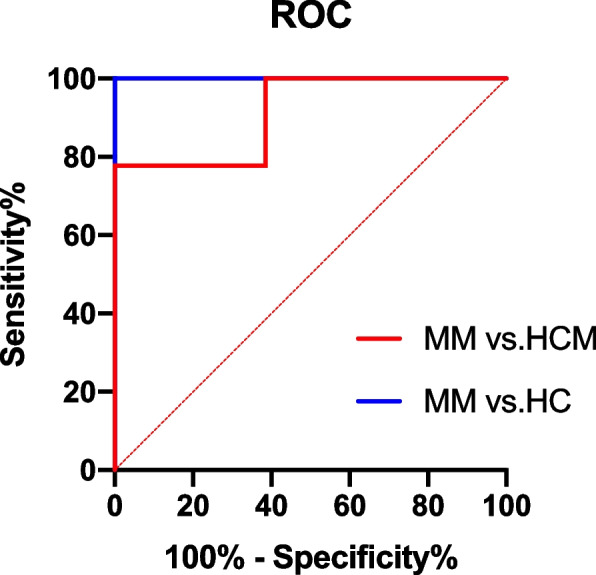
ROC curve of the native T1 value between MM group with healthy controls group (HC) and non-obstructive hypertrophic cardiomyopathy group (HCM)

ROC curve analysis of native T1 value between MM group with healthy controls group and non-obstructive hypertrophic cardiomyopathy group showed that the cut-off value of T1 were 1345 ms and 1348 ms, the areas under the curve were 1.0, *P* < 0.001and 0.91, *P* = 0.001, the specificity were 100% and 100%, and the sensitivity were 100% and 61.6% (Fig. 1), respectively.

**Fig. 2:**
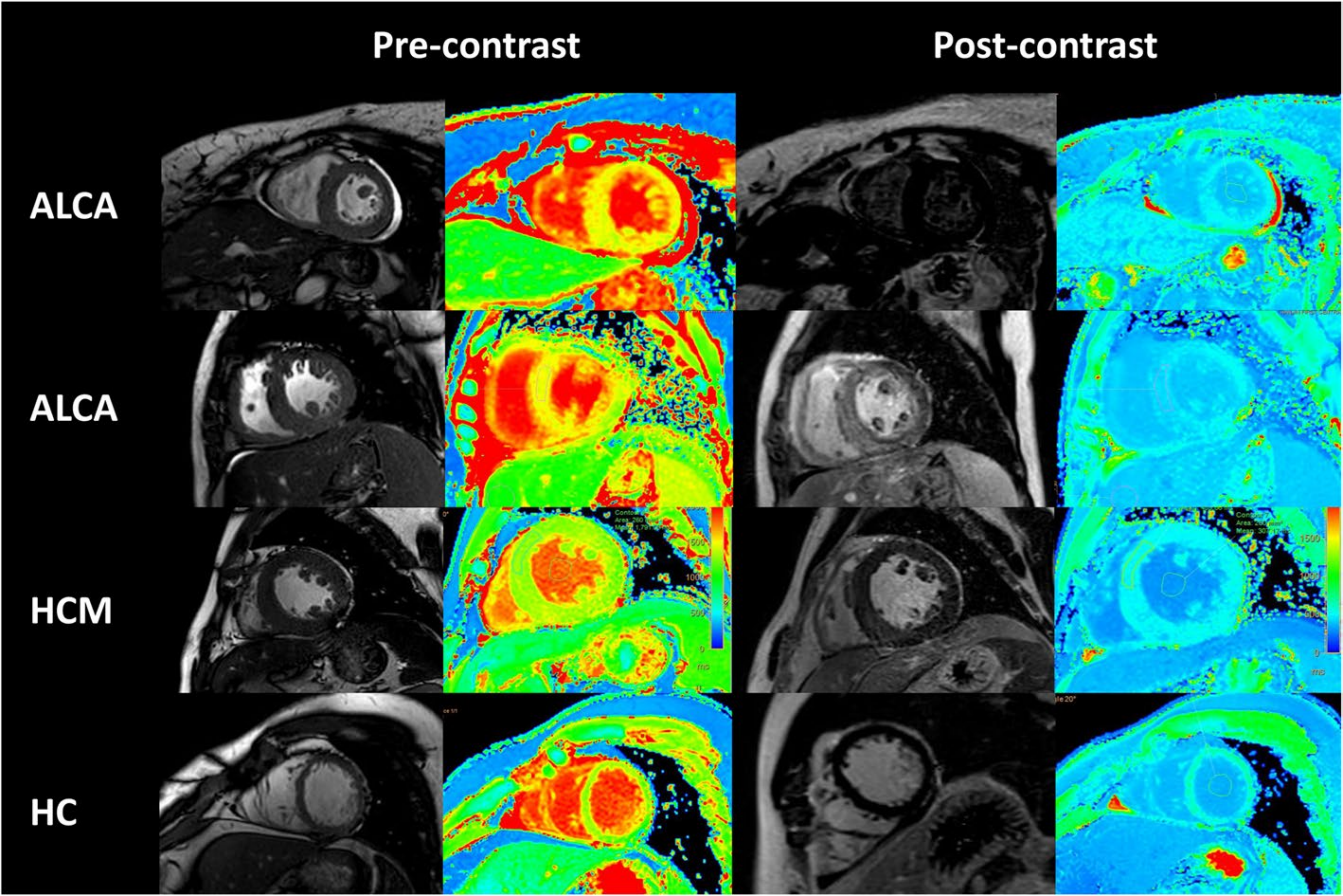
A short-axis slice showing in two MM patient amyloidosis involving myocardium, one HCM and one HC. AL CA concentric hypertrophy, thickening of the RV wall in second AL CA, and pericardial effusion on cine steady-state free precession, with a typical sub-endocardial pattern of delayed enhancement in first MM, while with a typical diffuse transmural pattern of delayed enhancement in second MM. Mean native T1 was elevated (T1 1552 ms and 1489 ms; normal range 1165–1289 ms for this sequence at 3 T), with a mean extracellular volume increased

Considering the systemic amyloidosis sedimentary characteristics and renal function in patients with conditions, there are three MM patients with amyloidosis involving myocardium after one period chemotherapy treatment (two months), had a review plain CMR scan, native T1 value is (1484.8 ± 6.5) ms after chemotherapy treatment and (1462.0 ± 71.3) ms before chemotherapy treatment, there was no statistically significant difference (*P* = 0.12), showed no obvious progress in amyloidosis involving myocardium. The images characteristics between MM group with healthy controls group and non-obstructive hypertrophic cardiomyopathy group showed in Fig. 2.

## Discussion

Myocardial thickening is a significant symptom of amyloidosis involving myocardium, characterized by diffuse thickening of the left ventricular wall, most frequently occurring when the left ventricular wall thickening of unexplained > 12 mm in the presence of suspected myocardial involvement [21]. In the study of 16 MM patients with amyloidosis involving myocardium, the left ventricular wall thickening (LVMT > 12 mm) occurred, and at this time, the myocardial compliance is reduced, which can lead to diastolic dysfunction, and in the late stage, systolic dysfunction can be caused, and some may be accompanied by pericardium and/or pleural effusion By including six cases with pericardial effusion and nine cases with pleural effusion, the emergence of effusion may be caused by cardiac dysfunction or amyloid deposition in pericardium or pleural effusion [22]. By amyloid deposition in the left ventricular wall, the thickness of the left ventricular wall changes first in morphology, the endocardium, endocardial fat and pericardial effusion during delayed enhancement can be observed onfat suppression sequencesand T2-weighted images.

The typical LGE model of amyloidosis involving myocardium can be characterized by diffuse subendocardial or transmural late enhancement pattern [23]. In this study, 16 MM cases all had LGE, 1. Although similar manifestations may occur in advanced ischemic heart disease, myocardial scarring in ischemic heart disease can be shown to be related to vascular distribution and myocardial thinning rather than myocardial thickening. Studies have shown that the myocardial enhancement patterns are positively correlated with amyloid deposition. The early stage of myocardial amyloidosis can appear LGE [24]. In addition to the performance of myocardial LGE, blood pool gadolinium (Gd) early clearance in amyloidosis patients is more obvious. Therefore, after injection of contrast medium 10 ~ 15 min, amyloidosis patients have a significantly lower signal intensity in blood pool than healthy subjects, signaling a much earlier reduction in blood flow to the heart cavity. This phenomenon is because systemic Gd contrast agents were stranded in the whole-body tissue amyloid [25]. Amyloidosis involving myocardium was a gradual accumulation of amyloid in the myocardial interstitium material, increasing ventricular wall thickness and mass and eventually causing cardiac diastolic and systolic dysfunction [26].

T1 mapping can detect cardiac involvement of amyloidosis and evaluate amyloid deposition in the heart. Native T1 mapping can measure T1 relaxation time of myocardium is of great value in evaluating interstitial dilation caused by myocardial edema and fibrosis. Post-contrast T1 mapping to distinguish whether T1 values for amyloidosis involving myocardium is also very meaningful. Krombach et al. [25], in a quantitative study of T1 mapping, demonstrated that when amyloidosis involving myocardium occurs, T1 values significantly increased. Although the ordinary T1WI scan on myocardial signal level is difficult to distinguish, the quantification of T1 value is helpful for diagnosis, T1 value threshold greater than or equal to 1273 ms (1.5 T), indicating amyloidosis, the diagnosis of myocardial amyloidosis has the highest accuracy, sensitivity of 84%, specificity of 89% and Post-contrast T1 mapping of myocardial T1 values significantly lower than normal. Amyloidosis involving myocardium in MM patientshas a poor prognosis, with an 8-month survival compared to a 4-year survival without myocardial involvement. Although the biopsy is the “gold standard” for diagnosing amyloidosis involving myocardium, it could not follow up on patients following treatment. CMR, except for early diagnosis and differential diagnosis, may be used to determine the severity of myocardial amyloid by assessing the extent of ventricular wall thickness, LVM, and LGE, as well as for follow-up to evaluate the effect of treatment and noninvasive examination [12, 27, 28]. For T1 mapping and LGE, Patients receiving treatment can be followed up longitudinally to evaluate the effect of treatment, identify cardiac involvement, and assess the duration of the patient's disease, and it is non-invasive [29].

### Limitation

In our study, the patient numbers were relatively small,and there are 9/26 HCM paitents admission for heart failure, the LVEF is lower than 20%, and have LGE; there are another 10/26 HCM paitents have LGE, the LVEF also lower; For single center cases may exist selection bias, it is one limitation, for future, more cases and multi-center cooperation are need. We enrolled the HCM group based on cardiac hypertrophy compared with experimental group.

## Conclusion

CMR examination showed MM’s cardiac amyloidosis enhancement pattern and measurement of myocardial ECV represents a possible noninvasive index of amyloid burden with potential utility for early diagnosis and disease monitoring. LGE joint T1 mapping wider clinical use techniques and follow-up the paitents’disease severity.

## Data Availability

The datasets used during the current study are available from the corresponding author on reasonable request.

## Abbreviations

MM: Multiple myeloma
CMR: Cardiac magnetic resonance
CA: Cardiac amyloidosis
LGE: Late gadolinium enhancement
HCM: Hypertrophic cardiomyopathy
HC: Healthy control
LVEF: Left ventricular ejection fraction
LVEDV: Left ventricular end-diastolic volume
LVESV: Left ventricular end-systolic volume
LVM: Left ventricular mass
ECV: Extracellular volume
